# Target Treatment Zones: A Zone‐Based Clinical Framework for Skin Quality Improvement Using Non–Cross‐Linked Hyaluronic Acid and Glycerol

**DOI:** 10.1111/jocd.71191

**Published:** 2026-09-18

**Authors:** Vorapot Siramangkhalanon, Harirak Artamnuayvipas, Kran Sophonpatima

**Affiliations:** ^1^ Hertitude Aesthetic Medical Bangkok Thailand; ^2^ The H Clinic Bangkok Thailand; ^3^ Face Gallery Clinic Bangkok Thailand

**Keywords:** aesthetic medicine, hyaluronic acid, skin quality, target treatment zones, visual aesthetic methodology

## Abstract

**Background:**

Aesthetic medicine has progressively shifted from volume toward skin quality as a key determinant of perceived age and facial attractiveness. Although non–cross‐linked hyaluronic acid–based injectables are widely used to improve skin hydration and appearance, clinical application remains largely technique‐driven, and structured decision‐making frameworks addressing skin texture and visual skin quality as distinct therapeutic objectives remain limited.

**Objective:**

To introduce target treatment zones (TTZs), a skin quality‐first, anatomy‐guided decision‐making framework within the visual aesthetic (VA) methodology, designed to support selective dermal intervention for texture refinement and patient‐perceived improvement in skin quality without altering facial volume or contour.

**Methods:**

Evidence on skin quality as a foundational aesthetic parameter was reviewed alongside the rationale for dermal texture refinement using non–cross‐linked hyaluronic acid combined with glycerol. The TTZ framework integrates anatomical zoning, zone‐specific clinical relevance, and biologically appropriate dermal depth selection. Illustrative clinical cases from routine aesthetic practice are included to demonstrate clinical feasibility, practical application, and conceptual understanding rather than objective efficacy.

**Results:**

The illustrative cases supported the practical application of the TTZ framework for identifying clinically relevant treatment zones and selecting localized dermal intervention while seeking to minimize overtreatment. Serial clinical observations suggested possible improvements in hydration, surface texture refinement, and visual skin quality without volumetric change or contour distortion, while changes in skin texture homogeneity, fine superficial lines, pore appearance, and perceived skin brightness were observed. No formal efficacy analysis was performed.

**Conclusion:**

TTZs are proposed as a conceptual clinical framework that may support the incorporation of skin quality enhancement as a structured planning step rather than as a solely technique‐dependent intervention. As an extension of the VA methodology, TTZs are intended to complement structural rejuvenation strategies and support natural, skin‐centered aesthetic outcomes.

## 1 Introduction

The focus of aesthetic medicine is shifting from an isolated correction of wrinkles and volume loss toward a comprehensive assessment of facial aging as a multilayered process [1]. While volumetric restoration and contour modification remain central to facial rejuvenation, these strategies alone do not fully address age‐related changes in skin texture, hydration, and visual quality. Skin quality is being increasingly recognized as an independent contributor to perceived age, facial attractiveness, and patient satisfaction [2, 3].

To meet this evolving standard of care, aesthetic therapies have advanced to target these specific physiological markers. Non–cross‐linked hyaluronic acid (HA)–based injectable formulations, commonly referred to as hydroboosters, are treatments that contain HA and other ingredients that deeply hydrate skin, smooth fine lines, and improve elasticity and visual properties by replenishing moisture and stimulating collagen production in the dermis. The primary mechanism of action of these injectable formulations extends beyond a simple volume augmentation to the fundamental modification of the skin's viscoelastic properties. These formulations, which may incorporate a combination of HA and glycerol, can function as a moisture depot, improving cell turgor and tissue tension [4]. At the cellular level, non–cross‐linked HA–based injectable formulations can act as bioactive scaffolds that attenuate the catabolic profile of aging skin. Chen et al. [5] demonstrated that a complex of low‐molecular‐weight sodium hyaluronate and its acetylated derivative can enhance Type I collagen accumulation in fibroblasts. Preclinical evidence suggests that HA complexes can downregulate matrix metalloproteinase‐1, the enzyme primarily responsible for collagen fragmentation in photoaged skin. Simultaneously, these complexes synergistically increase the secretion of Type I collagen, mitigating the age‐related dermal thinning [6].

At present, hydroboosters have seen use within the prejuvenation demographic: younger individuals seeking preventative treatments to delay the onset of aging [7]. A 2022 consensus of aesthetic practitioners across the Asia‐Pacific region established four specific emergent perceptual categories to standardize the definition of skin quality: skin tone evenness, skin surface evenness, skin firmness, and skin glow. These categories include measurable attributes such as pigmentation, redness, pores, fine lines, elasticity, and hydration [8]. Within this framework, the expert panel highlighted distinct regional preferences, noting that Asian patients tend to desire a more even and luminous skin appearance when compared with the preference of Caucasian patients. Uneven skin tone, uneven skin surface, skin laxity, as well as sebaceous gland hyperactivity and enlarged pores were identified as the most common skin quality issues encountered in clinical practice in Asia [8].

The visual aesthetic (VA) methodology was developed as a strategic framework for facial rejuvenation, concentrating on proportion, framework, sagginess, and projection as core analytical pillars [2]. Building upon this foundation, the present work introduces target treatment zones (TTZs) as a conceptual extension that specifically focuses on skin quality enhancement. TTZs aim to support structured, anatomy‐guided decision‐making for superficial dermal treatment, focusing on dermal texture and visual skin quality without altering facial volume or contour. At present, TTZs should be considered a conceptual clinical framework rather than a validated clinical guideline or standardized treatment protocol.

## 2 Skin Quality as a Foundational Aesthetic Parameter

### 2.1 Defining Skin Quality

Skin quality comprises several interrelated parameters, including surface texture homogeneity, fine superficial wrinkles, pore visibility, hydration status, elasticity, and radiance. Alterations in these parameters may occur early in the aging process and persist independently of volumetric or structural changes. Clinically, compromised skin quality often presents as reduced skin brightness, uneven texture, or a fatigued appearance, even in patients without significant contour deficiency [1].

From an aesthetic perspective, deterioration in skin quality can diminish an otherwise appropriate structural rejuvenation. Consequently, interventions targeting dermal hydration and texture refinement may play an essential role in both prejuvenation strategies for younger patients and rejuvenation approaches for older individuals.

### 2.2 Skin Quality Versus Volume and Contour

Volumetric correction primarily targets the deep anatomical layers, including skeletal support and fat compartments, to restore facial structure and contour [9, 10]. In contrast, skin quality‐focused interventions target the papillary and upper reticular dermis. While complementary within the VA methodology, these approaches have distinct therapeutic objectives and are not interchangeable [2, 9].

In patients with thin skin, reduced elasticity, or advanced dermal deterioration, reliance on volumetric correction alone may necessitate excessive filler use, increasing the risk of overcorrection and unnatural outcomes. Prioritizing skin quality enhancement in such cases may improve skin elasticity and firmness, reduce dependency on volume, and support more natural facial harmony.

## 3 Biological Rationale for Dermal Texture Refinement

### 3.1 Role of Non–Cross‐Linked HA

HA is a fundamental component of the extracellular matrix, contributing to dermal hydration, viscoelasticity, and tissue homeostasis. Unlike cross‐linked HA, non–cross‐linked HA is rapidly metabolized by endogenous hyaluronidases present in the skin, typically within a day [11]. Therefore, its lasting clinical benefits are derived from natural biological responses and improved hydration rather than a long‐term physical structure. When injected into the dermis, non–cross‐linked HA formulations integrate within the extracellular matrix to hold water without adding persistent volume [4, 11].

This increased matrix hydration significantly improves the dermal microenvironment beyond just passively boosting moisture. Fibroblasts may respond favorably to these enhanced extracellular conditions, which potentially support collagen homeostasis and dermal integrity without reliance on volumetric expansion [5, 12].

### 3.2 Dermal Depth Considerations

Within the TTZ framework, dermal delivery is specifically directed toward the papillary–upper reticular dermal interface, corresponding to an approximate depth of 1 mm. This plane provides access to a high density of metabolically active fibroblasts while potentially minimizing surface irregularities associated with overly superficial injections and avoiding volumetric effects seen with deeper placement [11].

### 3.3 Role of Glycerol in Dermal Hydration

Glycerol is a well‐established humectant with a recognized role in maintaining skin hydration and barrier function [13]. Within the dermal environment, glycerol attracts and retains water, supports epidermal barrier integrity, and reduces trans‐epidermal water loss [13]. When co‐administered with HA via microinjections, glycerol acts synergistically to stabilize interstitial fluid retention and provide a sustained dermal moisture depot [4]. In vivo biophysical evaluations have demonstrated that this injected combination significantly improves cell turgor, tissue tension, and the physiological environment of the extracellular matrix, leading to improvements in viscoelastic skin properties such as firmness and fatigue resistance [4]. When combined with non–cross‐linked HA, glycerol may also stabilize dermal hydration and contribute to enhancement of visual skin quality [14, 15].

## 4 TTZs: A Skin Quality‐First Framework

TTZs represent a skin quality‐focused extension of the VA methodology. Rather than functioning as an injection map or procedural protocol, TTZs are intended to provide a structured decision‐making framework that guides clinicians in determining where intervention to improve skin quality may be most relevant and why a specific region warrants prioritization.

### 4.1 Anatomical Organization

**FIGURE 1:**
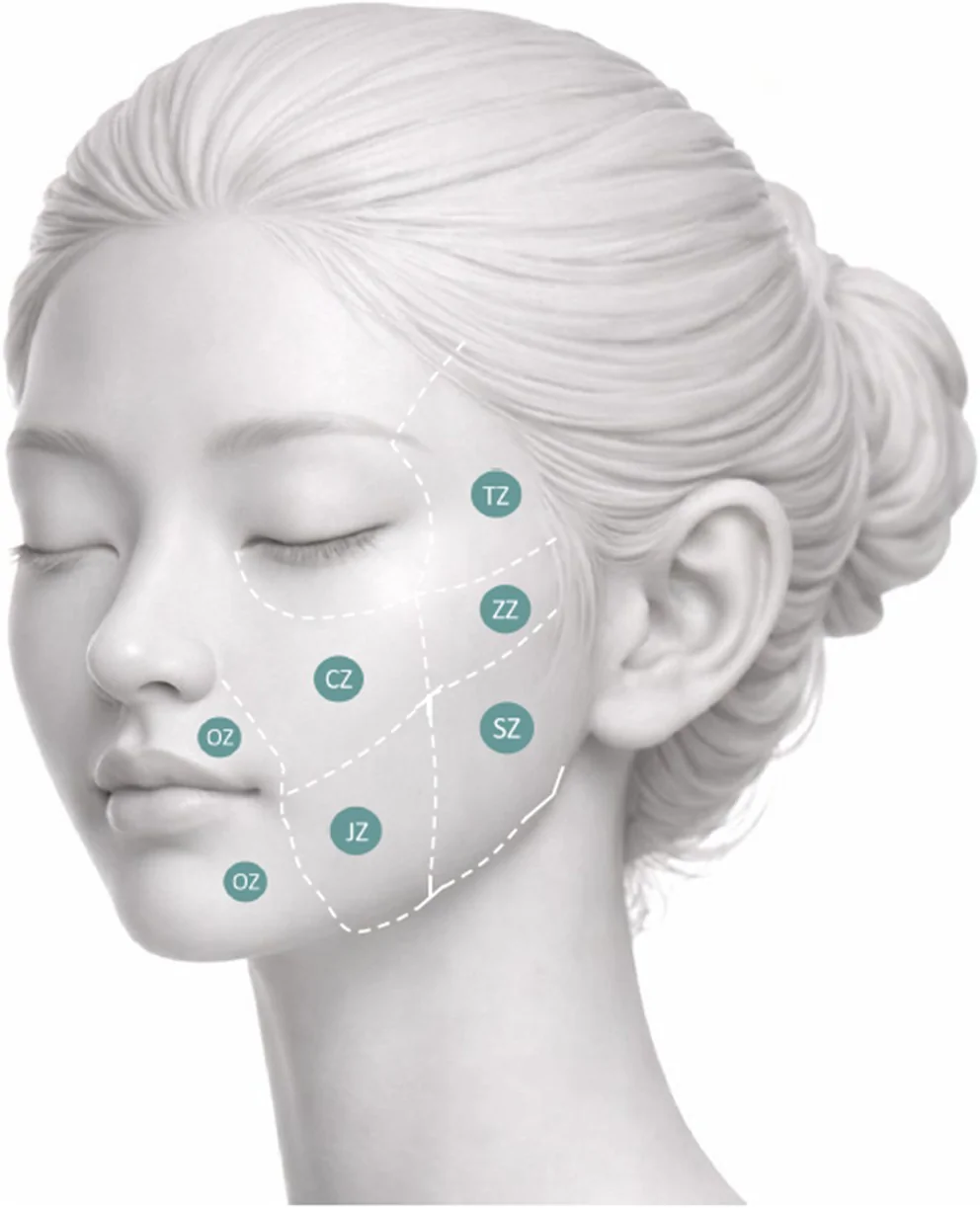
Schematic illustration of the target treatment zone framework, highlighting anatomically and functionally defined regions for aesthetic intervention. CZ, cheek & infraorbital zone; JZ, jowl zone; OZ, peri‐oral zone; SZ, sub‐zygomatic zone; TZ, temporal zone; ZZ, zygomatic zone.

The TTZ framework divides the face and neck into clinically relevant regions, including the frontal, temporal, zygomatic, sub‐zygomatic, cheek, jowl, and peri‐oral zones (Figure 1). Each zone is associated with distinct patterns of dermal aging, biomechanical behavior, and aesthetic relevance. The framework is adaptable and does not mandate intervention across all zones in every patient.

Within this framework, a zone is considered for skin quality‐focused intervention only when localized concerns are present, such as dryness, visibly dilated pores, fine or static superficial wrinkles, surface texture irregularities, including atrophic or acne scarring, or reduced visual skin quality. The presence of one or more of these features supports prioritization of the corresponding zone, independent of volumetric or structural considerations.

### 4.2 Clinical Objectives

The objective of TTZ‐guided intervention is to address localized impairments in skin quality by potentially improving surface homogeneity and dermal hydration, which may support enhancement of visual skin quality. For the purposes of the framework, high visual skin quality is defined by an increased light reflection and decreased shadowing resulting from improved surface homogeneity, minimized pore visibility, and attenuated fine lines. While currently assessed via standardized clinical photography and patient‐reported outcomes [2], future approaches should incorporate objective tools such as glossmeters, reflectometers, or polarized imaging systems to quantify these changes in surface reflectance. This framework is designed to complement, rather than replace, structural or volumetric rejuvenation strategies while preserving facial contour, proportion, and natural dynamics.

## 5 Clinical Application of the Framework

### 5.1 Study Design and Case Selection

The cases provided in this study are illustrative and descriptive in nature and are not intended to demonstrate clinical efficacy, establish comparative outcomes, or support protocol standardization. No statistical comparisons were performed. Cases were retrospectively selected from routine aesthetic practice to demonstrate the application of the framework in patients requiring targeted skin quality improvement. The primary selection criterion was the clinical presentation of localized skin quality impairments, including enlarged pores, reduced firmness, coarse surface texture, and fine superficial wrinkles. Additionally, the selected patients presented without clinically significant volumetric deficiency or facial laxity. This highlights the framework's focus on superficial dermal intervention rather than structural restoration.

### 5.2 Patient Selection

**FIGURE 2:**
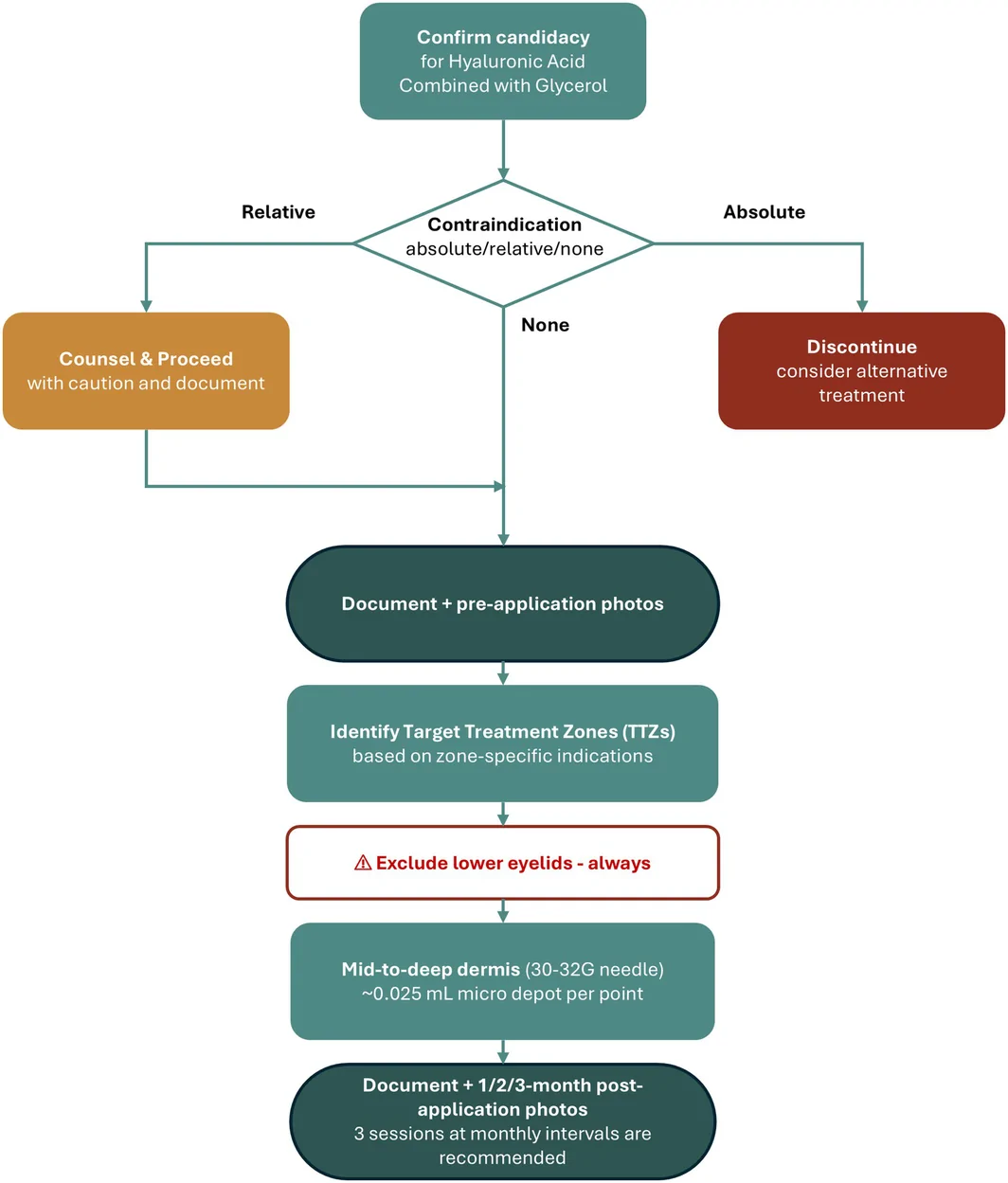
Decision‐making flowchart for daily‐practice selection of TTZs in hyaluronic acid–glycerol skin‐booster (hydrobooster) treatment. Patient candidacy is first confirmed, and C/I are classified as absolute, relative, or none. Absolute C/I warrants discontinuation of treatment with consideration of an alternative modality; relative C/I permits treatment following counseling, informed consent, and documentation. Baseline photographs are obtained before treatment. TTZs are then identified according to zone‐specific indication. The lower eyelid is excluded from treatment in all cases owing to thin dermal architecture and increased risk of edema and product visibility. The product is delivered into the mid‐to‐deep dermis using a 30–32‐gauge needle in approximately 0.025‐mL micro‐depots per injection point, distributed across the selected zone(s). Standardized follow‐up photographs are taken at 1, 2, and 3 months post‐application; three sessions at 4‐ to 6‐week intervals are recommended per study protocol. Diamond, decision point; teal, process step; dark teal, start/end; amber, proceed with caution; dark red, discontinue treatment; C/I, contraindications; TTZ, target treatment zone.

Patient selection should include assessment of treatment indications and relevant contraindications before TTZ identification and treatment planning. Proposed contraindications within the present framework are summarized in Table 1, while the proposed clinical decision‐making process for patient screening, TTZ selection, treatment, and follow‐up is summarized in Figure 2.

**TABLE 1:** Absolute and relative contraindications for hyaluronic acid–glycerol skin‐booster treatment within the TTZ framework.

Contraindication category	Contraindications
Absolute	Known hypersensitivity to hyaluronic acid or other product components; pregnancy and breastfeeding; active infection, inflammation, or skin lesion at the injection site, including acne flare, herpes outbreak, or dermatitis; active malignancy at or near the treatment site
Relative	Uncontrolled autoimmune disease or immunosuppressive therapy; bleeding disorders or anticoagulant/antiplatelet use, with a higher risk of bruising; history of keloid or hypertrophic scarring; unrealistic patient expectations

### 5.3 Framework Intervention Principles

**FIGURE 3:**
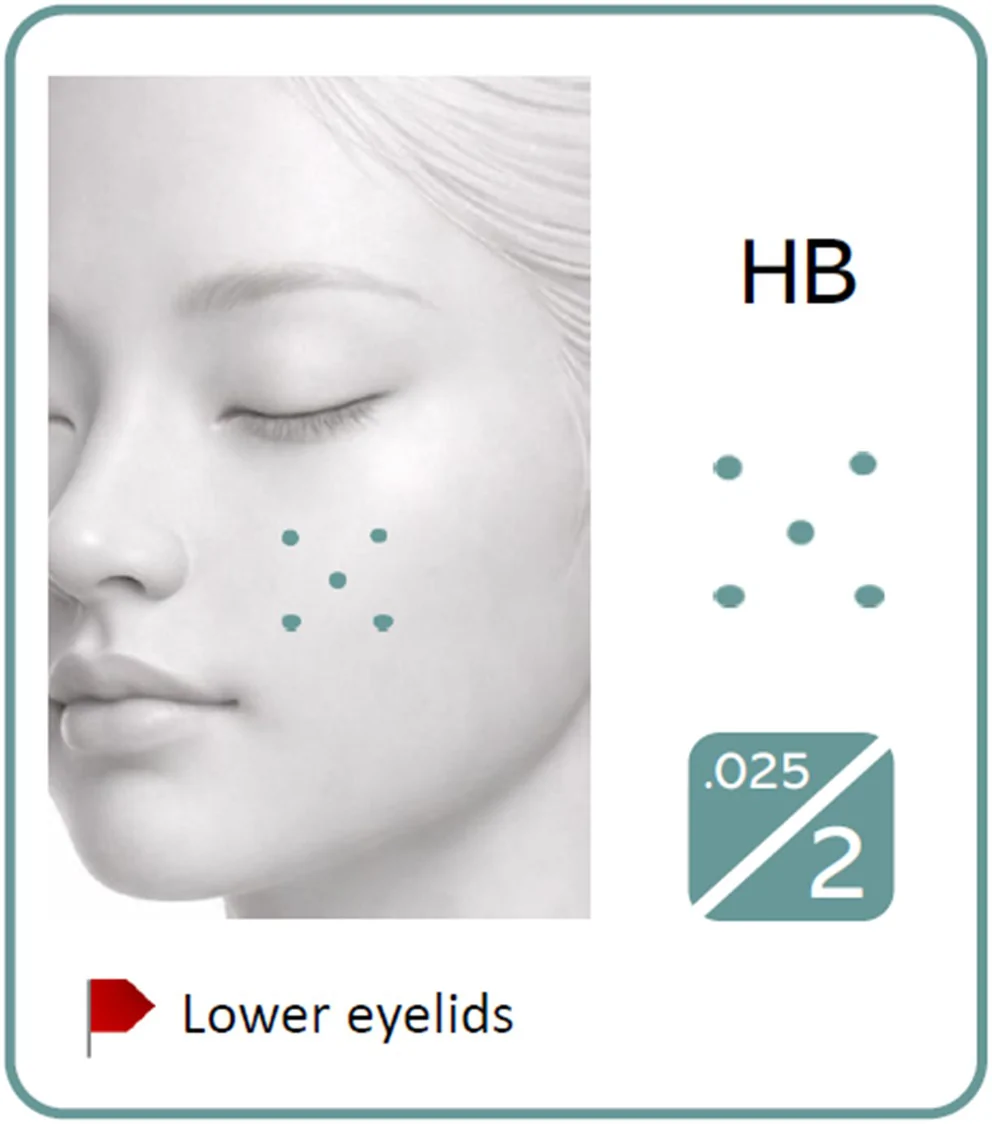
Schematic representation of HB injection into the target treatment zones. The product is administered into the mid‐to‐deep dermis using a 30–32 G sharp needle, delivering micro‐depots of approximately 0.025 mL per injection point in a distributed pattern. This technique is intended to enhance dermal hydration and improve surface skin quality without inducing a volumetric effect. The lower eyelid region is excluded due to its thin dermal architecture and higher susceptibility to edema and visibility of the injected product. HB, hydrobooster.

TTZs are intended to function as a conceptual planning layer within facial aesthetic assessment. Zone selection is guided by individual patient concerns, anatomical characteristics, and baseline skin quality rather than predefined treatment protocols. Clinical application focuses on biologically appropriate dermal targeting with pinpoint injection of the non–cross‐linked HA–based formulation into the TTZs (Figure 3) with the aim of supporting hydration and texture refinement without inducing volumetric change.

### 5.4 Illustrative Case Applications

#### 5.4.1 Case 1

**FIGURE 4:**
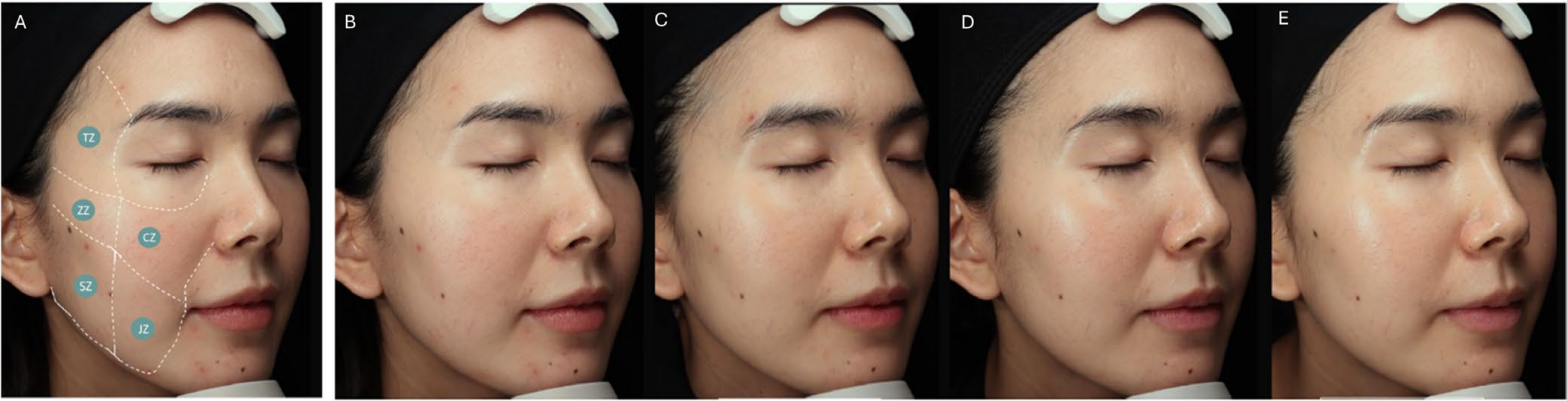
TTZs used in the treatment for a 29‐year‐old female as well as follow‐up improvements over 3 months. (A) TTZ map, (B) before treatment, (C) after 1 month, (D) after 2 months, and (E) after 3 months. CZ, cheek & infraorbital zone; JZ, jowl zone; SZ, sub‐zygomatic zone; TTZ, target treatment zone; TZ, temporal zone; ZZ, zygomatic zone.

A 29‐year‐old Thai female presented with enlarged pores, reduced skin firmness, coarse surface texture, and mild atrophic acne scarring, without facial laxity or structural volumetric deficiency (Figure 4). Selective superficial dermal intervention was performed according to the TTZ framework. Serial clinical observation suggested improved hydration, refinement of pore appearance, and progressive smoothing of surface texture while appearing to preserve facial contour and expression.

#### 5.4.2 Case 2

**FIGURE 5:**
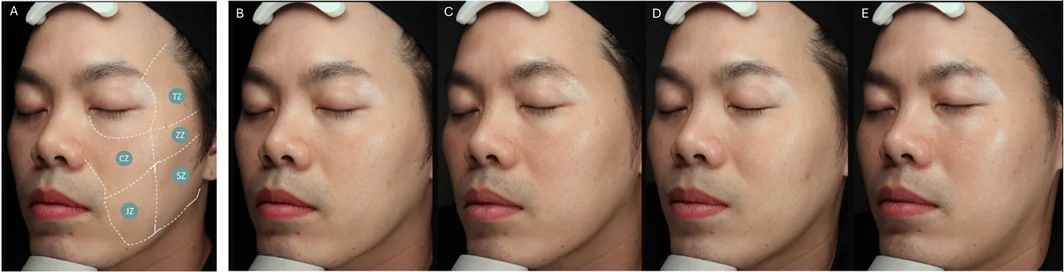
TTZs used in the treatment for a 25‐year‐old male as well as follow‐up improvements over 3 months. (A) TTZ map, (B) before treatment, (C) after 1 month, (D) after 2 months, and (E) after 3 months. CZ, cheek & infraorbital zone; JZ, jowl zone; SZ, sub‐zygomatic zone; TTZ, target treatment zone; TZ, temporal zone; ZZ, zygomatic zone.

A 25‐year‐old male presented with enlarged pores, fine periorbital lines, and reduced skin hydration associated with reduced visual skin quality, without structural volumetric deficiency (Figure 5). TTZ‐guided intervention was performed over three sequential sessions at monthly intervals. Serial clinical observation suggested attenuation of fine superficial lines, refinement of pore appearance, and gradual improvement in surface smoothness and perceived skin brightness over 3 months.

#### 5.4.3 Case 3

**FIGURE 6:**
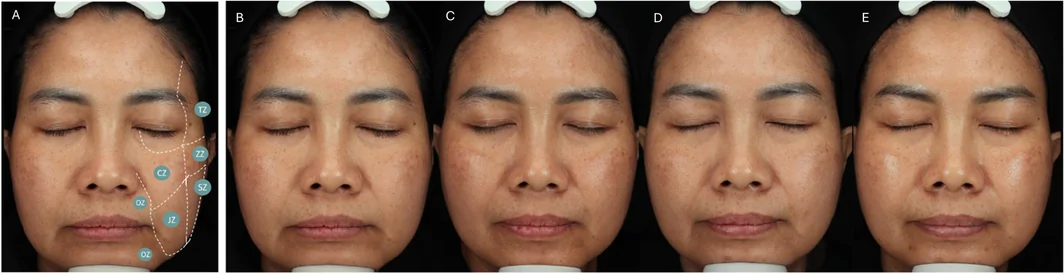
TTZs used in the treatment for a 52‐year‐old female as well as follow‐up improvements over 3 months. (A) TTZ map, (B) before treatment, (C) after 1 month, (D) after 2 months, and (E) after 3 months. CZ, cheek & infraorbital zone; JZ, jowl zone; OZ, peri‐oral zone; SZ, sub‐zygomatic zone; TTZ, target treatment zone; TZ, temporal zone; ZZ, zygomatic zone.

A 52‐year‐old Thai female presented with coarse skin texture, fine superficial wrinkles, and reduced visual skin quality without clinically significant volumetric deficiency (Figure 6). TTZ‐guided intervention was performed over three sequential monthly sessions. Serial clinical observation suggested progressive improvement in surface texture, fine wrinkles, and perceived skin brightness over a three‐month period, while facial contour and expression appeared to remain unchanged.

## 6 Discussion

Facial rejuvenation may not be fully achieved through volumetric correction alone. Skin quality deterioration independently contributes to perceived aging and patient dissatisfaction [1, 2]. Despite widespread use of dermal hydration therapies, a lack of structured planning frameworks has limited consistency and strategic integration.

TTZs are intended to address this gap by reframing skin quality enhancement as a methodological decision‐making step rather than a technique‐driven intervention. By emphasizing anatomical relevance, indication‐driven prioritization, and biologically appropriate dermal targeting, TTZs may facilitate integration effectively within the VA methodology and help support natural, balanced aesthetic outcomes. The novelty of the TTZ framework lies in structured zone prioritization and indication‐based treatment planning.

The descriptive changes observed in the illustrative cases are broadly consistent with established literature regarding the dual‐action mechanism of chemically non–cross‐linked HA complexes. Existing evaluations have highlighted the biphasic nature of these interventions, which consists of an immediate phase and a delayed phase [16, 17]. Initially, patients may experience an immediate hydration benefit perceived as a subtle plumping effect due to the hygroscopic properties of the injected matrix. Over time, this transitions into a delayed remodeling phase characterized by mechanical fibroblast activation and subsequent collagen stimulation, ultimately restoring elasticity and providing improved dermal firmness and elasticity. Further in vitro evidence supports this stimulatory effect. A 15% increase in fibroblast viability and a 9.7‐fold upregulation of Type I collagen gene expression were observed following exposure to an HA‐based formulation, confirming that non–cross‐linked HA can actively remodel the dermal extracellular matrix beyond passive hydration [18].

By applying the TTZ framework to target the papillary‐to‐upper reticular dermal interface, clinicians may seek to align treatment placement with the proposed biological effects of non–cross‐linked HA formulations, with the aim of supporting dermal hydration and longer‐term tissue quality rather than inducing volumetric change. These findings are consistent with a multicenter randomized controlled trial by Kerscher et al. [19], which evaluated a cohesive poly‐densified matrix HA‐glycerol formulation. At week 12, aesthetic improvements were observed in 90.7% of the patients, with significantly enhanced skin hydration and effects persisting for up to 9 months.

The application of this framework is particularly relevant for Asian facial morphotypes. As highlighted by regional consensus data, Asian patients frequently prioritize skin texture improvements, such as minimizing pore visibility and improving tone homogeneity, over structural alteration [7]. Furthermore, Asian morphotypes are often highly susceptible to undesirable volumetric widening when highly cross‐linked fillers are overutilized to address superficial textural concerns [20]. The thicker and more robust skin structures in the faces of East Asians require deeper filler injections for volumization [21], which reinforces the importance of stratification. TTZs are intended to target the superficial dermis for improvement of skin quality, a plane distinct from the sub‐superficial musculoaponeurotic system spaces used for volumetric correction [21]. For Asian patients with isolated textural concerns and no volume deficit, TTZ may offer a volume‐neutral option that avoids the risks associated with superficial placement of cross‐linked fillers. This separation of skin quality‐focusing interventions from volumetric correction could also allow clinicians to address dermal deterioration without risking undesirable volume changes, aligning with the regional preference for more volume‐neutral rejuvenation.

From a technical perspective, the TTZ framework is intended to provide a more structured approach to treatment planning, particularly in historically challenging anatomical regions. Areas such as the periorbital zone and tear trough are complex; indiscriminate superficial injections in these regions frequently result in persistent fluid retention, the Tyndall effect, or surface irregularities due to impaired lymphatic drainage [22, 23]. The anatomical zoning utilized in the TTZ framework helps guide the clinician to adjust product distribution, injection depth, and technique based on the specific biomechanical limitations of the zone. This structured approach may support clinical decision‐making and could help reduce the risk of adverse events in delicate areas by prioritizing anatomical knowledge over repetitive technique.

### 6.1 Limitations and Future Directions

The examples presented here have several limitations. First, they are primarily based on the authors' clinical experience and illustrative cases rather than controlled prospective studies; therefore, the findings are descriptive and are not intended to establish comparative efficacy. The absence of a control group and the lack of blinded evaluation also preclude such comparisons. Furthermore, the selection of cases may introduce single‐center expert practice bias, and the exclusive focus on a specific HA and glycerol formulation results in limited generalizability of product‐specific findings. Further clinical validation is required.

Second, outcome assessment relies largely on qualitative clinical evaluation, including improvements in skin texture, perceived skin brightness, and surface homogeneity. The absence of standardized quantitative measures such as validated skin quality scales, biophysical assessments, or imaging‐based analysis limits objective reproducibility.

Third, although TTZs provide a structured decision‐making framework, treatment outcomes remain dependent on the practitioner. Variability in technique, injection depth, and product distribution may contribute to heterogeneity in real‐world results.

Finally, long‐term durability and optimal maintenance strategies have not been systematically evaluated. The temporal dynamics of treatment response and cumulative effects require further investigation.

At present, TTZ remains a conceptual clinical framework and has not yet been validated as a clinical guideline or standardized treatment algorithm. Future prospective studies should evaluate the validity, reproducibility, and clinical utility of the TTZ framework using standardized assessment methods. Objective skin‐quality measurements could include instrument‐based evaluation of hydration, elasticity, trans‐epidermal water loss, surface topography, pore visibility, and skin reflectance, supplemented by standardized clinical photography and blinded evaluator assessment. Validated clinical rating scales and patient‐reported outcome measures should also be incorporated to assess perceived changes in skin texture, radiance, satisfaction, and treatment acceptability [1, 3]. Prospective cohort studies could initially examine within‐patient changes over time, whereas randomized or comparative studies could compare TTZ‐guided treatment with conventional technique‐driven approaches, untreated control areas, or alternative treatment‐planning strategies. Such studies would help determine whether TTZ‐guided planning improves consistency, treatment selection, patient‐reported benefit, and safety across different practitioners, populations, and anatomical zones.

Investigation of TTZ integration in multimodal approaches, such as fillers, threads, and energy‐based devices within the VA methodology, may further enhance treatment precision and outcomes. In addition, emerging digital technologies, including artificial intelligence‐assisted treatment planning, may improve personalization, consistency, and scalability of TTZ‐guided interventions.

## 7 Conclusion

TTZs are proposed as a potentially reproducible, conceptual, skin quality‐first decision‐making framework within the VA methodology intended to systematize existing clinical principles. By shifting the focus from technique‐dependent application to structured planning, TTZs may offer a scalable and adaptable approach to enhancing skin texture and visual quality, providing a foundation for future clinical and methodological investigation.

## Author Contributions

All authors contributed to case collection, data interpretation, manuscript drafting, and critical revision, and approved the final version. All authors take responsibility for the integrity of the work as a whole and have approved this version to be published.

## Funding

The authors have nothing to report. No external funding was received for the clinical cases or development of the framework. Medical writing and editorial support were funded by Menarini APAC.

## Ethics Statement

This study includes illustrative clinical cases derived from routine aesthetic practice. It does not involve experimental interventions or prospective data collection. All medical procedures described in this report were conducted in accordance with the Declaration of Helsinki.

## Consent

Written informed consent was obtained from all patients whose clinical photographs were used.

## Conflicts of Interest

V.S. is self‐employed in the Hertitude Clinic, Bangkok, Thailand. H.A. is self‐employed in The H Clinic, Bangkok, Thailand. K.S. is self‐employed in the Face Gallery Clinic, Bangkok, Thailand. V.S. serves as a consultant for A. Menarini (Thailand) and A. Menarini Asia‐Pacific. VA Methodology is an intellectual property belonging to V.S. No logistical or financial support for the execution of this work was received.

## Data Availability

The data that support the findings of this study are available from the corresponding author upon reasonable request.
